# Is Fitspiration the Healthy Internet Trend It Claims to Be? A British Students’ Case Study

**DOI:** 10.3390/ijerph18041837

**Published:** 2021-02-13

**Authors:** Maria Limniou, Charlotte Mahoney, Megan Knox

**Affiliations:** Department of Psychology, University of Liverpool, Liverpool L69 7ZA, UK; ckmahoney29@gmail.com (C.M.); meganknox98@hotmail.com (M.K.)

**Keywords:** fitspiration, Instagram, body satisfaction, mood satisfaction, state self-esteem

## Abstract

The increasingly popular #fitspiration community on Instagram aims to promote body positivity and inspire health in its followers. However, fitspiration accounts often endorse unattainable, overly fit body ideals. The aim of this study is to explore the effects of viewing fitspiration photos on body image and fit-ideal internalisation. We compared 109 British students’ (18–50 years-old) responses on state self-esteem, mood satisfaction, body satisfaction and fit-ideal internalisation before and after viewing fitspiration photos. Online questionnaires exposed students to either five male or five female fitspiration photos, respectively for their given gender. Photos were sourced from public Instagram accounts. This study also examined the influence age and Instagram usage have on body image. Exposure to fitspiration photos produced a significant reduction in state self-esteem, mood satisfaction and fit-ideal internalisation, but had no significant influence on body satisfaction. Age had no effect on body image; however, gender impacted mood satisfaction and fit-ideal internalisation. Instagram usage influenced fit-ideal internalisation, with specific Instagram factors, such as how the importance of a photo’s “likes” were negatively associated with state self-esteem, mood and body satisfaction. Unexpectedly, Instagram frequency use and posting were related to higher levels of state self-esteem. Detailed explanations of the findings and potential future research opportunities are also discussed.

## 1. Introduction

Historically, in Western countries, a major focus has been placed on the concept of body image, whereby physical appearance equates to attractiveness [1]. Shape and weight, both factors regarded to be in the control of the individual [2], are at the core of body dissatisfaction and eating disorders [3]. For example, unattainable cultural ideals of thin and slender women [4] have been positively portrayed across different media platforms, including television programmes, films and advertisements [5]; subsequently creating negative body images [1]. Social media has also facilitated the interaction of diverse behaviours, making it easier for role models to impart societal concepts of body ideals [6,7]. Initially the body image research was focused on Facebook use, since this was the first social media platform [8,9]. More detailed examinations of Facebook have deemed that it was not merely the duration of time an individual spends on the site, but the amount of time spend on photo-related activities that correlate with body image concerns [10]. Recently, another Social Networking Site (SNS), Instagram, has placed larger emphasis on photo-related activities (i.e., sharing and “liking” photographs/videos). “Likes” have become a form of social reinforcement, where individuals may view the number of “likes” received as direct evaluative feedback about attractiveness and self-worth [11]. Instagram usage, such as frequency of posting and time spent viewing photos, has positively correlated to body image concerns [12,13]. The increased interaction and exposure to unhealthy body ideals has consequently impacted body satisfaction [14] and mental health [15]. The hashtag (#) has also been used by Instagram users to post and search images in communities such as #fitspiration or #fitspo.

Fitspiration, an amalgamation of the words “fitness” and “inspiration”, has been put forward as a healthy antidote to thinspiration [16]. Thinspiration, an unhealthy internet-based trend, idealises and promotes bodily thinness [17], through harmful weight loss strategies [6]. The newer emerging trend of fitspiration, however, has focused on inspiring individuals to achieve an empowered body image through engaging in a healthier lifestyle [18]. The legitimacy of highly unregulated fitspiration content [19] and its focus on weight loss alongside idealising a thin, yet toned, muscular physique provides cause for concern. Content analyses of fitspiration media found that it conforms to sociocultural ideals of physical attractiveness [20]. Despite fitspiration’s aims to inspire positivity and a healthy lifestyle, most research to date suggests that its exposure leads to a lower mood and decreased body dissatisfaction [15,21,22], except for one study, which found no effects [23]. However, fitspiration photos solely focus on obtaining one body shape, a thin-toned physique [24]. The absence of differing body shapes creates body dysmorphia for individuals whose body image does not resemble the popularised physique [21]. Fitspiration is advocated by Instagram “influencers”, role models who display inspiring photos, alongside motivational texts and healthy food suggestions [25]. The ubiquity of fitspiration photos means exposure is inevitable [24], as over 65 million posts on Instagram acuminate fitspiration messages [18], therefore encroaching the lifestyle onto countless Instagram users. Theoretically, it is conceivable to believe that fitspiration messages have a progressive influence on health and wellbeing [6]. However, recent research has revealed that fitspiration photos correlate with a decline in mental health [15], as there is a disregard for personal and structural bodily differences. It appears that body image dissatisfaction results from an accumulation of frequent, small-sized exposures to fitspiration images [17]. Fitspiration messages influence followers to look like themselves, although the followers might have different factors such as health issues, different body types and lifestyles, that make it impossible to look like certain influencers. This, alongside low trait self-esteem, could lead to a sense of failure if they follow all the guidance of fitspiration but do not see the same results as their influencers.

The Tripartite Influence Model (TIM) [26] is a theoretical approach developed to incorporate many of the variables hypothesised to influence body image. The model suggests that body image development arises from three formative influences: parents, peers, and media; and is mediated by two factors, excessive appearance comparison and internalisation of cultural ideals. In the case of fitspiration media, the ideal internalised would be that of an athletic (“fit”) body. In the framework of this model, through internalisation and comparison, negative body image is a by-product of unrealistic cultural messages from the fitness accounts on Instagram. TIM has high levels of empirical support from the literature as an explanation of how sociocultural pressures link to body image disturbance [27,28]. However, previous research indicates that societal pressure and exposure to thin and muscular ideals in media predict increased likelihood of internalisation [29,30,31], but has neglected study into internalisation of the “fit” ideal shown in fitspiration imagery. As a combination of the thin and muscular ideals [16], fitspiration may be internalised in a similar manner and presents a new and potentially damaging threat to body-esteem.

Most social media and body image research studies have been focused on female behaviours [32]. However, a male ideal body image portrayed as hyper-muscular physiques may also cause depression, lower self-esteem, and disordered eating behaviours in male audiences [33]. Notably, until recently, muscular and fit-ideal physiques were solely desired by males, whilst women favoured a thin ideal [16]. Therefore, little research has focused on the association between fit-ideal internalisation and fitspiration photos, in a mixed-gendered sample. Previous studies have also assumed that different genders desire different body ideals [25], instead of apprehending the possibility that all genders internalise identically. The media encompasses Generation Z’s purpose [34] with most of their waking day spent on social media. Considering this, fitspiration photos have been shown to vastly influence the self-esteem of younger generations [15,33], highlighting the influence that age has on social media usage. Previous research supports an age-related reduction in social comparisons [35] and has found that older adults report a lower tendency for comparison than younger adults. Furthermore, younger adults have been found to use SNS more often than older adults [36]. Therefore, it could be suggested that with increased SNS use and a greater affinity to compare oneself to others, younger adults will be more negatively affected by viewing fitspiration media portraying unattainable body ideals than older adults.

In summary, the present study aimed to explore the effects of fitspiration exposure on a university student population (males and females), focusing on body image within the fit-ideal internalisation framework. The influence fitspiration photos have on body image was examined under the prevalence of state self-esteem, body satisfaction, mood satisfaction and fit-ideal internalisation. Specifically, the objectives of this study were to explore:

1. The difference in fit-ideal internalisation after fitspiration exposure.
2. The association between gender, age and Instagram usage, with state self-esteem (SSE), mood satisfaction, body satisfaction and fit-ideal internalization.
3. The association between Instagram usage factors (the number of Instagram followers, the number of people followed, the number of photos posted, the frequency of Instagram use and the importance of a photo’s “likes”) with SSE, mood and body satisfaction and fit-ideal internalisation.

## 2. Materials and Methods

### 2.1. Participants and Experimental Conditions

A sample of 109 British Instagram users from a first-year undergraduate psychology cohort of a UK Northwest University aged between 18 and 50 years (M = 20, SD = 3.3) were recruited. Ninety-four (86.20%) of them were females, with the remaining 15 (13.80%) being males. The response rate for students who fully completed this study questionnaire was 31% for the first-year psychology cohort. The ratio of the females who participated in this study was 31.3% out of the whole first-year psychology female cohort and 29.4% for males. The participants satisfied a VanVoorhis and Morgan (2007) [37] conservative rule of thumb, where 7 participants are necessary per condition, with 50 participants overall.

Participants were recruited over a two-month period (December 2019–January 2020) after the study gained an ethical approval from the university Ethics Committee. Specifically, an online questionnaire was distributed via an anonymous link to undergraduate psychology students through University social media (e.g., Facebook and Instagram) and through an internal recruitment scheme, using opportunity sampling as part of the recruitment process. A participant information sheet and consent form were provided prior to the questionnaire and participants received a debrief sheet after questionnaire completion. Participants could anonymously withdraw at any time based on a unique number, without providing any reason. Online questionnaires, developed on the web-secured Qualtrics survey platform, were the same for both males and females, except for the body images. When the participants selected their gender choice, five different Instagram images were revealed to them. The fitspiration photographs were sourced from a public Instagram #fitspiration area. A different set of images was shown to participants based on their gender. The first four of the fitspiration images in each gender group illustrated females or males posed in fitness clothing in a gym environment. The fifth image depicted a female or a male engaged in a fitness activity in a gym environment. The travel images were the same for the two gender categories and were sourced from a public Instagram #travel area, illustrating images from various travel destinations including natural landscapes, but without including any physical appearance.

The study employed a within-subject design, with levels of the independent variable (travel and fitspiration), to assess the effect of fitspiration imagery on body image factors and internalisation. Furthermore, a correlational design was used to assess the relationship between Instagram usage factors, age and gender with body image and internalization. Data were analysed using within-subject ANOVAs, two-step hierarchical multiple regressions and simple regressions.

### 2.2. Questionnaire

Demographic information (i.e., age, gender and nationality) and Instagram usage (i.e., use frequency, post frequency, “likes” importance, numbers of followers and following numbers) items were included in the first part of the questionnaire. The second part of the questionnaire included 5 travel photographs sourced from public Instagram “travel” hashtag profiles followed by the 20-item State Self-esteem Scale (SSE) [38], 5-item mood satisfaction scale, 5-item body satisfaction scale and 11-item Fit-ideal Internalisation Scale (adapted version of Sociocultural Attitudes Towards Appearance Scale-3 -SATAQ-3) [39]. Finally, the last part of the questionnaire included the 5 #fitspiration photographs of individuals posing in fitness clothing and/or completing exercise. The fitspiration photographs were chosen to provide a representative overall image of that displayed in everyday Instagram use. The questionnaire then provided the same scales-SSE, mood satisfaction, body satisfaction and fit-ideal internalization in the same order as previously. The mood and body satisfaction scales were inspired by Heinberg and Thompson (1995) [40]. Overall, the questionnaire had 98-items and took approximately 20 min to complete.

## 3. Results

A within-subject ANOVA statistical analysis was conducted to compare participants’ responses on SSE, mood satisfaction, body satisfaction and fit-ideal internalisation before and after their exposure to fitspiration photos (Table 1). Overall, there was not a significant effect on body satisfaction, but there was a significant effect on state self-esteem, mood satisfaction and fit-ideal internalisation scores after participants were exposed to fitspiration photos.

**Figure 1:**
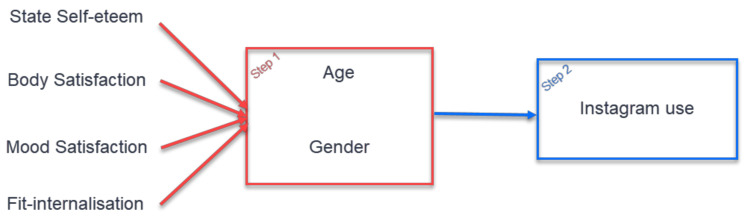
The two-step regression statistical analysis which includes age and gender in first step and Instagram use in the second step.

A correlational design was used to assess the relationship between Instagram usage, age and gender with body image and internalisation. Specifically, a two-step hierarchical regression was conducted to assess the association between age, gender and Instagram factors with SSE, mood and body satisfaction and internalization. Body image was considered under the prevalence of State Self-Esteem, body satisfaction, mood satisfaction and fit-ideal internalisation. Age and gender were entered at Step 1, followed by Instagram use at Step 2 (Figure 1).

A multiple regression was run to predict state self-esteem from age, gender and Instagram use. At Step 1, age and gender predicted approximately 7% of variance in self-esteem, though no significant variables were produced. The inclusion of Instagram use, at Step 2, lead to a non-significant increase in variance accounted for by the model. The overall regression model predicted 8% of variance in self-esteem (adjusted R^2^ = 0.08; F (3,105) = 2.94, *p* = 0.037). The hierarchical regression indicated that age (β = 0.035, *p* = 0.136), gender (β = 0.363, *p* = 0.131) and Instagram (β = −0.056, *p* = 0.527) use did not influence self-esteem.

A multiple regression was run to predict body satisfaction from age, gender and Instagram use. At Step 1, age and gender predicted approximately 6% of variance in body satisfaction, with no significant variables produced. The inclusion of Instagram usage at Step 2 lead to a non-significant increase in variance accounted for by the model. The overall regression model predicted 6% of variance in body satisfaction (adjusted R^2^ = 0.06; F (3,105) = 2.13, *p* = 0.101). Therefore, the hierarchical regression indicated that age (β = 0.060, *p* = 0.216), gender (β = 0.745, *p* = 0.132) and Instagram use (β = −0.040, *p* = 0.824) do not significantly influence body satisfaction.

A multiple regression was run to predict mood satisfaction from age, gender and Instagram use. At Step 1, age and gender predicted approximately 9% of variance in mood satisfaction. Gender, unlike age (β = 0.075, *p* = 0.163), was found to be a significant predictor of mood satisfaction (β = 1.203, *p* = 0.022). The inclusion of Instagram use at Step 2 lead to a non-significant increase in variance accounted for by the model. At this step, all variables non-significantly impacted mood satisfaction. The overall regression model predicted 9% of variance in mood satisfaction scores (adjusted R^2^ = 0.09; F (3,105) = 3.61, *p* = 0.016). The hierarchical regression showed that when considering age and gender as factors that influence mood satisfaction, gender has a significant impact. However, when including Instagram use, all variables have no significant effect on mood satisfaction.

A multiple regression was run to predict fit-ideal internalisation from age, gender and Instagram use. At Step 1, age and gender predicted approximately 24% of variance in fit-ideal internalisation. Gender, unlike age (β = 0.005, *p* = 0.461), was found to be a significant predictor of fit-ideal internalisation (β = 0.451, *p* < 0.001). The inclusion of Instagram use at Step 2 lead to a significant increase in variance accounted for by the model. At this step, gender (β = 0.377, *p* < 0.001) and Instagram use (β = −0.081, *p* = 0.015) had a significant impact on fit-ideal internalisation, while age was not a significant predictor (β = 0.002, *p* = 0.790). The overall regression model predicted 28% of variance in fit-ideal internalisation scores (adjusted R^2^ = 0.28; F (3,105) = 13.47, *p* < 0.001). The hierarchical regression showed that gender and Instagram use have a significant influence on fit-ideal internalisation.

**Figure 2:**
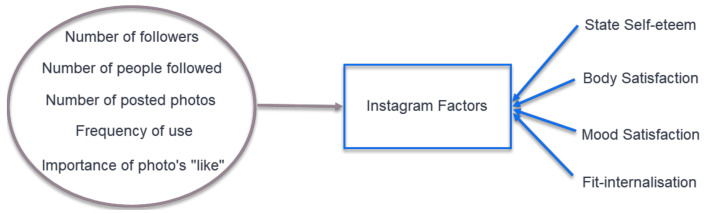
Simple regression statistical analysis between Instagram factors and state self-esteem, body satisfaction, mood satisfaction and fit-internalisation.

Simple regressions were also conducted to further understand the influence that specific Instagram use has on body image after exposure to fitspiration photos. Five specific Instagram factors were considered: the number of Instagram followers, the number of people followed, the number of photos posted, the frequency of Instagram use and the importance of a photo’s “likes” (Figure 2).

A simple regression investigated the influence Instagram factors have on state self-esteem (SSE). The regression model predicted approximately 17% of variance in state self-esteem (Adjusted R^2^ = 0.17, F (5,103) = 5.49, *p* < 0.001). Although the number of followers (β = −0.10, *p* = 0.520) and followings (β =0.10, *p* = 0.482) were not significantly associated with SSE, there was a strong positive association between Instagram use frequency (β= 0.22, *p* = 0.021) and post frequency (β = 0.26, *p* = 0.012) with SSE. There was also a strong negative association between the importance placed on “likes” (β = −0.30, *p* = 0.002) with SSE.

Additionally, a simple regression investigated the influence Instagram factors have on body satisfaction. The regression model predicted approximately 8% of variance in body satisfaction (Adjusted R^2^ = 0.08, F (5,103) = 2.84, *p* = 0.019). The importance placed on “likes” (β = −0.22, *p* = 0.029) had a significant negative correlation with body satisfaction. Usage (β = −0.17, *p* = 0.080), post frequency (β = 0.17, *p* = 0.126), the number of followers (β = −0.12, *p* = 0.445) and the number individuals followed (β = 0.16, *p* = 0.270) were not associated with body satisfaction.

Furthermore, a simple regression investigated the influence Instagram factors have on mood satisfaction. The regression model predicted approximately 17% of variance in mood satisfaction (Adjusted R^2^ = 0.17, F (5,103) = 5.44, *p* < 0.001). The importance of “likes” (β = −0.41, *p* < 0.001) had a significant negative influence on mood satisfaction. Usage (β = −0.16, *p* = 0.091), post frequency (β = 0.08, *p* = 0.428), the number of followers (β = −0.002, *p* = 0.988) and the number individuals followed (β = 0.13, *p* = 0.331) were not associated with mood satisfaction.

The final simple regression investigated the influence Instagram factors have on fit-ideal internalisation. The regression model predicted approximately 21% of variance in fit-ideal internalisation (Adjusted R^2^ = 0.21, F (5,103) = 6.82, *p* < 0.001). Fit-ideal internalisation was a negative significant influence by the importance of “likes” (β = −0.37, *p* < 0.001) and the frequency of Instagram use (β = −0.20, *p* = 0.028). Post frequency (β = −0.02, *p* = 0.830), number of followers (β = −0.16, *p* = 273) or number of accounts individuals followed (β = 0.13, *p* = 0.330) were not associated with fit-ideal internalisation.

## 4. Discussion

Fitspiration imagery is extensively documented and frequently used across multiple social media platforms [25]. Due to conflicting research, it is unclear as to whether fitspiration has detrimental consequences on audiences [41]. However, body image is a multi-dimensional, subjective representation of one’s body [42], with body satisfaction being one of its major components. Therefore, it appears that fitspiration images may affect some areas of an individual’s representation of themselves more than others, making its impact potentially less damaging than previously suggested. However, the current study suggests that even a small amount of exposure can negatively influence state self-esteem. Participants only viewed five fitspiration images; if extended out to the exposure one would come across during day-to-day Instagram use [17], the potential harm may become huge. These results coincide with the Tripartite Influence Model’s theoretical stance of media providing an influential pressure of unrealistic societal body ideals on its users, and supports previous research concluding that fitspiration exposure reflects an accumulation of frequent, small-sized insults to one’s esteem [17].

Within the framework of internalisation, the findings of this study contradict expectation. Participants’ levels of internalisation decreased after viewing the fitspiration images. This disputes the model’s view of exposure to ideals leading to internalisation and causing adverse effects. Since exposure to fitspiration did have negative effects on individuals’ mood and state self-esteem, there must be mechanisms other than internalisation which occur upon viewing body image ideals. The literature supports the influence of appearance comparisons in mediating the relationship between fitspiration images on body image development [15]. Therefore, it would be reasonable to suggest the empirically supported appearance comparison aspect of the model [32,43] may play a more prominent role in body image development. Furthermore, unlike the current study, fitspiration images on Instagram are posted alongside guilt-focused captions encouraging restrictive appearance standards [44]. Therefore, perhaps for individuals to internalise the fit-ideal, they must be exposed to both images and messages promoting the “fit” body.

It was expected that individuals with a higher use of Instagram and investment in “likes” would internalise the fit-ideal more. However, the study’s results showed an opposite relationship, with individuals having lower fit-ideal internalisation than less frequent Instagram users or those who did not find “likes” as important. This contradicts the expectation and provides no supporting evidence that an increased likelihood of exposure to fit-ideals or buying into the importance of one’s photos being socially reinforced and accepted (“liked”) leads to fit-ideal internalisation. Additionally, there were no associations between Instagram use with body or mood satisfaction, post frequency with mood satisfaction, body satisfaction and fit-ideal internalisation or the number of followers and followings with SSE, mood satisfaction, body satisfaction and fit-ideal internalisation.

However, a key factor brought about in the results was that of investment in “likes” being associated with numerous negative effects, including lower levels of state self-esteem, mood satisfaction and body satisfaction. This adds to the findings of Tiggemann, Hayden, Brown and Veldhuis (2018) [45] who found that higher investment in “likes” was associated with increased facial dissatisfaction. In both the current study and Tiggemann et al. (2018) [45], extreme investment in “likes” was related to dissatisfaction in a way that overall Instagram use was not; therefore, this factor may be especially problematic for extensive users of Instagram. This finding carries the potentially impactful practical application that individuals who use Instagram should be actively discouraged from seeking “likes” on their posts and should not equate the number of “likes” to their attractiveness or self-worth. This message could be added to media literacy which proves beneficial in reducing the impact of SNS use on body image concerns [46,47].

Following Facebook research [10], Instagram frequency of use and increased posting activity would be correlated with adverse effects. However, in the current study, those who used Instagram more and posted more frequently had a higher state of self-esteem after fitspiration exposure than those who were not avid users. Although this finding was somehow unexpected, some researchers have also found more positive effects in relation to individuals’ self-views [48]. It appears that intentionally revealing certain information about oneself on an online profile, such as posting photographs, may benefit self-esteem [49]. Through careful selection, users tend to present positive self-representations on their profiles [50]. Photographs on Instagram are shared and saved permanently, and therefore a rich source of self-evaluative information is always available. Research into self-affirmation shows that through evaluation of self-presented information, users’ self-reflective processes are activated, and individuals are made aware of the positive features of their self, leading to increased self-esteem [51,52]. Individuals view the use of SNSs as preferable compared to other online activities in order to boost their self-esteem, especially after a self-esteem threat [53]. A self-esteem threat could be in the form of damaging upward comparisons to fitspiration images. It therefore makes sense that in the current study, those with a higher Instagram use would not suffer from as low a state of self-esteem after viewing fitspiration images, for their self-affirmation has led to a more positive overall self-view.

Findings of this investigation showed that there were no associations between age and any of the variables. Although the sample size of this study is small, there is evidence that the inverse relationship between age and Instagram use might not support the findings of previous research [36]. Furthermore, the findings do not support the theory of weakened social comparisons in older adults [35] leading to poorer body image. Furthermore, gender was not a predictor of state self-esteem or body satisfaction. However, even though males internalised the fit-ideal more, females were found to have a lower mood satisfaction. This is an important evidence as it was predicted that since fitspiration images are described as a combination of both thin and muscular ideals [18], males and females would be affected in the same manner. The muscular and thin ideals have different effects of internalisation and related body image issues depending on gender [33,54] and the current results provide evidence for similar gendered effects of fit-ideals. The fit-ideal therefore is not necessarily an equal combination of the thin and muscular ideals and should potentially be treated as a new ideal, independent of others. Regarding the practical applications of this finding, media literacy could be adapted for male and female specific needs in relation to the differing effects of fitspiration exposure, protecting against the damage of social media appearance ideals in women but not in men [55]. By making media literacy more specific by gender, it would become even more effective at reducing the detrimental influence of fitspiration on both a male and female population.

Whilst findings give insight into how Instagram portrayals of the body can impact self-perceptions, including body image and self-esteem, the current study has some limitations. For example, the sample was relatively small and restricted to British students from only one UK University. For that purpose, this study is unpowered to provide future interventions on this area and it should be welcomed from the scientific audience as a pilot study in the fitspiration area, with potentially valuable findings. Reflecting the population of psychology undergraduates at the University, the sample had a female gender bias and used adults of a similar age (M = 20, SD = ±3.3). For a fuller, more comprehensive picture of the effects if fitspiration media, future research should investigate groups of individuals other than University students, expanding on the size and typology of the sample. For example, research could investigate the effects of Instagram fitspiration on a younger population, who are increasingly using social media platforms [56], and who will be exposed to fitspiration earlier in life. As childhood is a developmental process in which multiple body-related beliefs begin [42], it is fundamental to conduct more longitudinal investigations with a wider ranging population to critically analyse the effects of fitspiration, and to establish what educational matters need to be taught to minimise the chances of young people experiencing body dissatisfaction. The present study mimicked a scrolling method to view photos, as shown on Instagram, but limited contact, as participants were unable to like or comment on the photo, both key features of Instagram [13]. Therefore, the study was unable to completely capture the complexity of using the Instagram application. Perloff (2014) [57] argues that interaction on social media separates the platform from other methods of mass media. Therefore, in future research a consideration of these key features is recommended, as they will provide an accurate representation of Instagram for participants, whilst also improving the reliability of interpreted results. Finally, qualitative research can be employed to allow a discussion of the influential features of social media and its impact on body image.

Previous literature supports the damaging role of internalisation due to consumption of thin and muscular ideals in media [29,30,31]. Since the current study found evidence for the opposite effect, it appears that the newer emerging fit-ideal may in fact be viewed entirely differently to both ideals. Furthermore, the current findings may also be due to the lack of captions alongside each image. Since fitspiration did not lead to an internalisation of the fit-ideal, future research should address the differences in consumer’s views and opinions of the fit-ideal compared to the thin and muscular ideals. Research could also focus more on fitspiration content, instead of solely viewing images. This could lead to a better understanding of fitspiration’s negative effects on those who encounter it.

## 5. Conclusions

This study explores the participants’ responses after exposing them to various Instagram fitspiration images based on their gender. There is not a similar research study that the authors are aware of which explores the role of fitspiration, state self-esteem, body satisfaction and body mood with Instagram factors under the same experimental conditions for females and males. In addition, most previous studies have focused on females only. Another interesting part regarding this study is related to the design process which explored participants’ responses on the same variables after their exposure to travel and fitspiration images. This study assumed that Instagram users may not only view fitspiration images but also various other different Instagram images over the same short period of time. Therefore, the evidence of this pilot study may expand on the notion that fitspiration exposure has a negative effect on the way individuals feel about themselves in relation to their self-esteem and mood. However, fitspiration was not found to be damaging to individuals’ body satisfaction, nor did it lead to an internalisation of the fit-ideal as previously suggested. Furthermore, the fit-ideal affected both males and females, but to different degrees, with females experiencing more mood dissatisfaction and males internalising the fit-ideal more. Therefore, the fit-ideal appears to be a different ideal-type than thin or muscular ideals and is not necessarily an equal combination of the two. Investment in “likes” was negatively associated with all body image factors and fit-ideal internalisation. This specific social aspect of Instagram appears to be especially alarming for body image development. Although the results appear overall to be quite negative, the study did find some unexpected positive results in the form of Instagram use and how posting can be related to higher levels of self-esteem. This suggests that there is potentially some benefit in the use of the fast-growing online platform on users’ self-confidence. Ultimately, expanding on the existing literature, the study found an overall negative effect of fitspiration media and investment in “likes” on users’ body image, though Instagram use has the potential to increase state self-esteem. Fitspiration photos have a negative influence on body image, thus making it imperative that exposure to artificial imagery on social media is limited. Apart from researchers, various other professional sectors such as psychologists and nutritionists along with sport and media specialists may gain practical insights from this study. For example, in order to promote a more effective health and fitness campaign, they may include a more diverse range of bodies along with idealised images, which may prevent people counting on “likes” to gain attractiveness as this study suggests. These professional sectors may also campaign and/or produce media programmes which will increase peoples’ awareness of fitspiration imagery-enhancing self-control, and as such reduce their temptation to compare themselves with others.

## Figures and Tables

**Table 1:** Means and Standard Deviation values for SSE, mood satisfaction, body satisfaction and fit-ideal internalisation, before and after fitspiration exposure.

	Before	After	ANOVA
State Self-Esteem (SSE)	3.15(±0.69)	3.06(±0.79)	F(1, 108) = 9.18, *p* = 0.003, np^2^ = 0.078
Mood Satisfaction	5.50(±1.73)	5.19(±1.84)	F(1, 108) = 13.68, *p* < 0.05, np^2^ = 0.112
Body Satisfaction	5.11(±1.57)	4.99(±1.61)	F(1,107) = 2.39, *p* = 0.125, np^2^ = 0.022
Fit-ideal Internalisation	1.43(±0.31)	1.39(±0.33)	F(1, 108) = 8.48, *p* = 0.004, np^2^ = 0.073

## Data Availability

The data that supported the findings of this study are available from the corresponding author, upon reasonable request.
